# Myocardial and mitochondrial effects of the anhydrase carbonic inhibitor ethoxzolamide in ischemia‐reperfusion

**DOI:** 10.14814/phy2.15093

**Published:** 2021-11-21

**Authors:** Alejandro Ciocci Pardo, Luisa F. González Arbeláez, Juliana C. Fantinelli, Bernardo V. Álvarez, Susana M. Mosca, Erik R. Swenson

**Affiliations:** ^1^ Centro de Investigaciones Cardiovasculares ¨Dr Horacio E Cingolani¨ CCT‐CONICET Facultad de Ciencias Médicas Universidad Nacional de La Plata La Plata Buenos Aires Argentina; ^2^ Department of Medicine, Pulmonary and Critical Care Medicine VA Puget Sound Health Care System University of Washington Seattle Washington USA; ^3^ Present address: Department of Biochemistry Membrane Protein Disease Research Group University of Alberta Edmonton Alberta T6G 2H7 Canada

**Keywords:** carbonic anhydrase, ethoxzolamide, ischemia‐reperfusion, mitochondria, myocardium, p38MAPK, PKCε

## Abstract

We have previously demonstrated that inhibition of extracellularly oriented carbonic anhydrase (CA) isoforms protects the myocardium against ischemia‐reperfusion injury. In this study, our aim was to assess the possible further contribution of CA intracellular isoforms examining the actions of the highly diffusible cell membrane permeant inhibitor of CA, ethoxzolamide (ETZ). Isolated rat hearts, after 20 min of stabilization, were assigned to the following groups: (1) Nonischemic control: 90 min of perfusion; (2) Ischemic control: 30 min of global ischemia and 60 min of reperfusion (R); and (3) ETZ: ETZ at a concentration of 100 μM was administered for 10 min before the onset of ischemia and then during the first 10 min of reperfusion. In additional groups, ETZ was administered in the presence of SB202190 (SB, a p38MAPK inhibitor) or chelerythrine (Chel, a protein kinase C [PKC] inhibitor). Infarct size, myocardial function, and the expression of phosphorylated forms of p38MAPK, PKCε, HSP27, and Drp1, and calcineurin Aβ content were assessed. In isolated mitochondria, the Ca^2+^ response, Ca^2+^ retention capacity, and membrane potential were measured. ETZ decreased infarct size by 60%, improved postischemic recovery of myocardial contractile and diastolic relaxation increased P‐p38MAPK, P‐PKCε, P‐HSP27, and P‐Drp1 expression, decreased calcineurin content, and normalized calcium and membrane potential parameters measured in isolated mitochondria. These effects were significantly attenuated when ETZ was administered in the presence of SB or Chel. These data show that ETZ protects the myocardium and mitochondria against ischemia‐reperfusion injury through p38MAPK‐ and PKCε‐dependent pathways and reinforces the role of CA as a possible target in the management of acute cardiac ischemic diseases.

## Graphical abstract

Ethoxzolamide (ETZ) limits the infarct size and postischemic myocardial dysfunction. ETZ attenuates the postischemic damage of the mitochondrial state and dynamic. p38MAPK‐ and protein kinase C epsilon‐dependent pathways are involved in the cardioprotection by ETZ. ETZ appears as a new pharmacology agent against reperfusion injury.

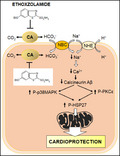

## 1 INTRODUCTION

During ischemia, cardiomyocytes accumulate H^+^ that activate isoform 1 of Na^+^/H^+^ exchanger (NHE1) and the Na^+^/${\text{HCO}}_{3}^{‐}$ (NBC) symporter and lead to an increased intracellular Na^+^ concentration (Garciarena et al., 2013; Vaughan‐Jones et al., 2006). This increase in intracellular Na^+^ concentration reduces the transmembrane Na^+^ gradient causing the Na^+^/Ca^2+^ exchanger to operate in a “reverse mode,” which leads to cytosolic accumulation of Ca^2+^. Both transporters (NHE1 and NBC) are functionally linked to carbonic anhydrase (CA) forming transport metabolons (Alvarez et al., 2005). CA is an enzyme that catalyzes rapid reversible interconversion of carbon dioxide and water into carbonic acid, H^+^, and ${\text{HCO}}_{3}^{‐}$ facilitating transmembrane fluxes of those ions in conjunction with others to regulate intracellular pH (Morgan et al., 2007).

Cardiac ventricular myocytes express several CA isoforms, which reside in the cytosol (CA II), mitochondria (CA V), sarcolemma (CA IV, CA XIV), and sarcoplasmic reticulum membrane (CA IV, CA IX, CA XIV) (Väänänen et al., 1991; Scheibe et al., 2006). These CAs are inhibited by several classes of compounds, such as sulfonamides, sulfamates, and sulfamides, among others. These compounds are used in the therapy of edematous states, glaucoma, hydrocephalus, acute mountain sickness, epilepsy, and obesity and cancer (Kumar et al., 2021). The efficacy of CA inhibitors sometimes depends on their diffusibility, which determines their accessibility or not to intracellular isoforms of CA.

At the cardiovascular level, there are data showing anti‐hypertrophic (Alvarez et al., 2007) and anti‐ischemic actions (Vargas et al., 2016) of CA inhibitors. Regarding this last effect, we recently demonstrated (Ciocci Pardo et al., 2018) that CA inhibition with the poorly diffusible CA inhibitor, benzolamide, decreases infarct size and improves the postischemic recovery of myocardial function. In a recent publication, we explored some of the downstream mechanisms responsible for the cardioprotection afforded by benzolamide (González Arbeláez et al., 2018).

These results *prima facie* indicate the important contribution of CA isoforms located on the sarcolemmal membrane with activity projected extracellularly and highlights the lack of knowledge about the participation of intracellular CA isoforms in the ischemic myocardium. Therefore, our interest was directed to determine if a highly diffusible inhibitor of CA, such as ethoxzolamide (ETZ), could have more benefit than only targeting extracellular CA isoforms.

In this respect, mitochondrial CA activity might affect permeability transition pore (mPTP) opening and the disturbance of mitochondrial dynamics, especially excessive mitochondrial fission, and thus contribute to ischemia‐reperfusion injury (Maneechote et al., 2017; Ong et al., 2015). Both effects can be triggered by the increase in intracellular Ca^2+^ concentration, which is also involved in other detrimental intracellular effects such as the activation of calcineurin phosphatase (Lakshmikuttyamma et al., 2003).

It was also previously reported that several mitogen‐activated protein kinases (MAPKs) such as p38MAPK and protein kinase C (PKC) participate in the pathways of cardioprotection (Feng et al., 2018; Heusch, 2015) against reperfusion injury.

Therefore, our aim was to study the effects of ETZ on myocardial and mitochondrial alterations following acute global ischemia and reperfusion in the isolated rat heart, analyzing the participation of p38MAPK‐ and PKCε‐mediated signal transduction pathways in the protection against ischemia‐reperfusion injury.

## 2 MATERIALS AND METHODS

### 2.1 Ex vivo heart preparation

All experiments were performed in accordance with the *Guide for Care and Use of Laboratory Animals* published by the National Institutes of Health (NIH Pub. No. 85‐23, revised 2011) and approved by the Animal Welfare Committee of the Faculty of Medicine, XXX1.

Wistar male rats of 4–5 months old (300–350 g) were anesthetized with an intraperitoneal injection of 25% urethane (0.6 ml/100 g). Central thoracotomy and heart excision were performed immediately after phase III anesthesia was reached, verified by the loss of pedal withdrawal reflex. Isolated hearts were perfused by the non‐recirculating Langendorff technique with Ringer's solution, buffered at pH 7.4, and maintained at 37°C. The heart was paced at 280 ± 10 beats/min. A latex balloon was placed into the left ventricle and filled with water to provide an end‐diastolic pressure (LVEDP) of 8–12 mmHg. Coronary flow was 11 ± 2 ml/min and coronary perfusion pressure was adjusted to approximately 60–70 mmHg. Left ventricular pressure (LVP) was acquired using an analog‐to‐digital converter and acquisition software (Chart V4.2.3 AD Instruments).

### 2.2 Protocols

**FIGURE 1:**
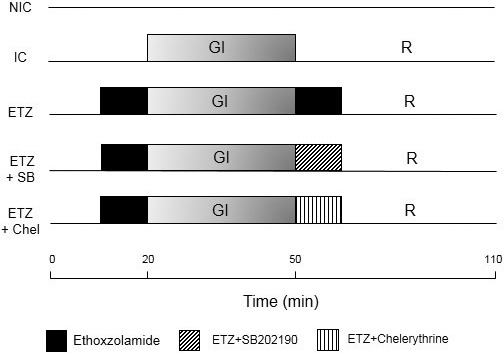
Scheme of experimental protocols. ETZ, ethoxzolamide; ETZ + Chel, ethoxzolamide plus PKC inhibitor; ETZ + SB, ethoxzolamide plus p38MAPK inhibitor; GI, global ischemia; IC, ischemic control; NIC, nonischemic control; R, reperfusion

After stabilization (20 min) the following experimental protocols were carried out (Figure 1):

1. *Nonischemic control (NIC*, *n* = *8)*: During 90 min the hearts were perfused with control solution.
2. *Ischemic control (IC) (n* = *7)*: Ischemia was induced by complete cessation of coronary flow (global ischemia) for 30 min. After that, the hearts were reperfused for 60 min.
3. *ETZ (n* = *7)*: The CA inhibitor ETZ at a concentration of 100 μM in the perfusion fluid was administered 10 min before ischemia and during the first 10 min of reperfusion. The dose selected was able to reduce the pHi recovery in heart papillary muscles submitted to an acid load, to a similar percentage to that observed after benzolamide (Ciocci Pardo et al., 2018). This action was associated to CA inhibition by ETZ.
4. *ETZ* + *SB (n* = *6)*: A quantity of 10 μM of SB202190 (p38MAPK inhibitor) and ETZ were co‐administered during the first 10 min of reperfusion.
5. *ETZ* + *Chel (n* = *6)*: A quantity of 1 μM of chelerythrine (PKC inhibitor) and ETZ were co‐administered during the first 10 min of reperfusion.

Other groups of hearts were subjected to the same protocols (*n* = 5 for each one) and then used for western blotting and for studies on isolated mitochondria.

### 2.3 Infarct size delineation

Infarct size was assessed by the triphenyl tetrazolium chloride (TTC) staining technique. At the end of reperfusion, left ventricle (LV) was frozen and cut into six transverse slices, which were incubated for 15 min at 37°C in a 1% solution of TTC. After, the slices were maintained in a solution of 10% formalin for 24 h and then they were weighed and scanned. Infarct size was calculated following the method described by Suzuki et al. (2001) and expressed as a percentage of total area (area at risk).

### 2.4 Myocardial function

Systolic function was assessed by the left ventricular developed pressure (LVDP) and maximal rise in velocity of LVP (+dP/dt_max_). Diastolic function was evaluated by the LVEDP.

### 2.5 Western blotting

A portion of LV was homogenized in ice‐cold RIPA buffer and centrifuged at 12,000 *g* for 15 min at 4°C. The protein concentration was evaluated by the Bradford method using bovine serum albumin as a standard. Sixty micrograms of supernatant proteins were resolved on SDS‐PAGE and transferred to PVDF membrane (2 h). The transference was confirmed by Ponceau S staining. Membranes were blocked with 5% nonfat milk and probed overnight at 4°C with antibodies against P‐p38MAPK (1:1000, Santa Cruz Biotechnology), p38MAPK (1:1000, Santa Cruz Biotechnology), P‐PKCε (1:1000, Santa Cruz Biotechnology), PKCε (1:1000, Santa Cruz Biotechnology), calcineurin Aβ (1:1000, Santa Cruz Biotechnology), P‐HSP27 (1:500, Santa Cruz Biotechnology), PSer637 Drp1 (1:1000, Cell Signaling), and Drp1 (1:1000, Santa Cruz Biotechnology). Membranes were washed four times prior to addition of anti‐mouse (1:5000, Lobov) or anti‐rabbit (1:5000, Sigma‐Aldrich) secondary antibody and protein bands were analyzed by a chemiluminescent system. GAPDH or total (phosphorylated plus non‐phosphorylated forms) protein expression was used as housekeeping protein.

### 2.6 Mitochondria isolation

Rat heart mitochondria were obtained by differential centrifugation (Mela & Seitz, 1979).

#### 2.6.1 Ca^2+^‐mediated mPTP opening

The resistance of mPTP to opening was assessed by the addition of 200 μM of CaCl_2_ to isolated mitochondria samples. If mPTP is open, solutes will be free to enter the inner matrix, causing the mitochondria to swell. These changes were observed as a light scattering decrease (LSD) and expressed as the difference between measures of LSD before and after Ca^2+^ addition (Baines et al., 2003).

#### 2.6.2 Calcium retention capacity

Calcium retention capacity (CRC) was defined as the amount of Ca^2+^ required to initiate triggering of massive Ca^2+^ release by isolated cardiac mitochondria (Obame et al., 2008). Extramitochondrial Ca^2+^ concentration was recorded with calcium green‐5N with excitation and emission wavelengths set at 506 and 532 nm, respectively. At the end of the preincubation period (300 s), successive pulses of 10 µM Ca^2+^ were added. When mitochondria cannot hold any more Ca^2+^, mPTP opening occurs and the concentration of the released ion increases in the incubation medium. CRC is expressed as nmol CaCl_2_/mg protein.

#### 2.6.3 Mitochondrial membrane potential

Mitochondrial potential changes were evaluated by measuring rhodamine 123 (RH 123) fluorescence quenching in a buffer containing RH‐123 0.1 μM. As suggested by Emaus et al. (1986), the experimental work was performed by exciting RH‐123 at 503 nm and detecting the fluorescence emission at 527 nm. Mitochondrial membrane potential (∆Ψm) was calculated following the instructions previously detailed (Scaduto & Grotyohann, 1999) using the Nernst–Guggenheim equation.

### 2.7 Statistical analysis

Data are presented as representative, as individuals experiment and as mean ±SD. Differences between groups were analyzed using one‐ or two‐way ANOVA with Turkey´s post‐test. A *p* < 0.05 was considered significant.

## 3 RESULTS

### 3.1 Measurements in the isolated rat heart

**FIGURE 2:**
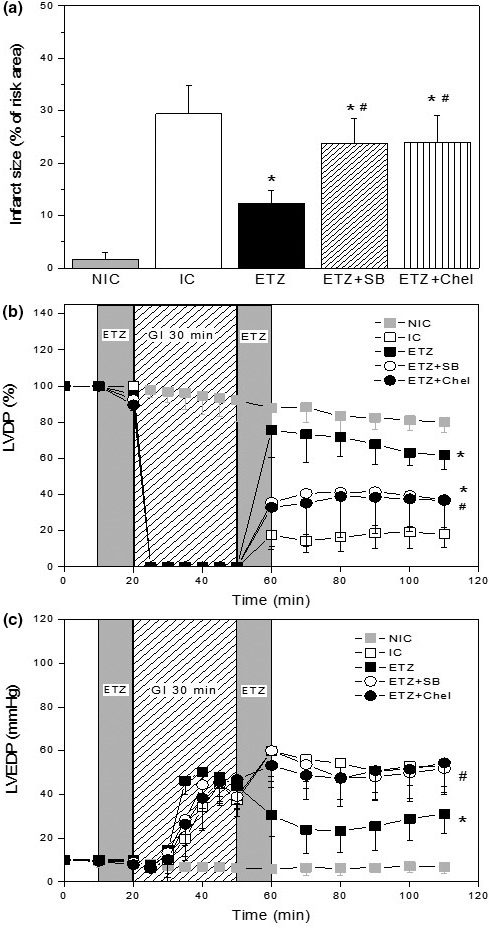
Mean data of infarct size (a), left ventricular developed pressure (LVDP, b), and left ventricular end‐diastolic pressure (LVEDP, c) of nonischemic (NIC, *n* = 8) and ischemic control (IC, *n* = 7) hearts and in hearts treated with ETZ (*n* = 7), ETZ + SB (SB202190, p38MAPK inhibitor, *n* = 6), and ETZ + Chel (chelerythrine, PKC inhibitor, *n* = 6). ETZ treatment decreased the infarct size and improved the postischemic recovery of myocardial systolic and diastolic functions observed in IC hearts. The infusion of ETZ in the presence of p38MAPK or PKC inhibition attenuated the cardioprotection afforded by ETZ. *p < 0.05 versus IC; ^#^ *p* < 0.05 versus ETZ. ETZ, ethoxzolamide; PKC, protein kinase C

The infarct size and the systolic and diastolic functions are shown in Figure 2. In untreated hearts, a period of 30‐min ischemia and 60‐min reperfusion produced an infarct size of approximately 30% of risk area. The administration of ETZ prior to ischemia and at the beginning of reperfusion reduced the infarct size to approximately 12%.

An improvement in postischemic recovery of systolic and diastolic functions was observed in ETZ‐treated hearts. During reperfusion, LVDP was greater and LVEDP was lesser than compared to that observed in IC hearts. Thus, LVDP was ~60% of pre‐ischemic value at the end of reperfusion. A similar pattern was observed when +dP/dt_max_ was analyzed. ETZ attenuated the increase in contracture, which increased to LVEDP values of ~30 mmHg at 60 min of reperfusion. The inhibition of p38MAPK with SB202190 (SB) or PKC with chelerythrine (Chel) abolished the beneficial effects observed in ETZ‐treated hearts. ETZ + SB and ETZ + Chel hearts have similar values of infarct size (Figure 2a), LVDP (Figure 2b), and LVEDP (Figure 2c) to those detected in IC group.

**FIGURE 3:**
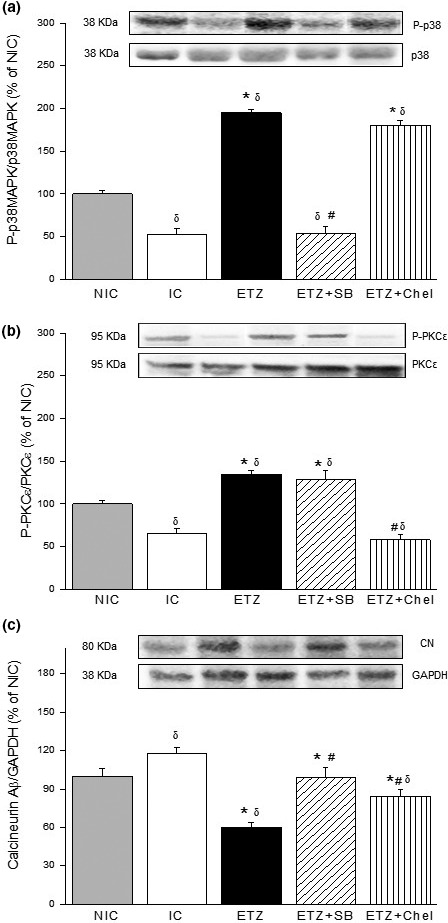
Representative immunoblots and summary of densitometric data of phospho‐p38MAPK/p38MAPK (a), phospho‐PKCε/PKCε (b), and calcineurin/GAPDH (c) ratios in nonischemic (NIC, *n* = 5) and ischemic control (IC, *n* = 5) hearts and hearts treated with ETZ (*n* = 5), ETZ + SB (*n* = 5), and ETZ + Chel (*n* = 5). Note that, the increase in the content of P‐p38MAPK and P‐PKCε produced by ETZ was abolished when those kinases were inhibited. The expression of calcineurin decreased by ETZ treatment and opposite changes were observed when ETZ was administered in the presence of p38MAPK or PKC inhibitor. ^δ^ *p* < 0.05 versus NIC; *p < 0.05 versus IC; ^#^ *p* < 0.05 versus ETZ. ETZ, ethoxzolamide; PKC, protein kinase C

The expression of phosphorylated forms of p38MAPK, PKCε, and calcineurin Aβ is shown in Figure 3. In IC hearts, the content of P‐p38MAPK and P‐PKCε was less than that of NIC group. ETZ treatment increased the level of these kinases reaching values significantly higher than those observed in NIC hearts. The hearts treated with ETZ plus SB showed a decrease in P‐p38MAPK expression without a change in P‐PKCε expression (Figure 3b). Similarly, treatment with ETZ in the presence of Chel caused a decrease in P‐PKCε expression without altering P‐p38MAPK content. Therefore, the inhibition of p38MAPK and PKC annulled the protective effect produced by ETZ. Opposite effects of ETZ on calcineurin Aβ content were detected. The level of this phosphatase increased in IC hearts and this rise was abolished by ETZ, but this was not observed when ETZ was administered in the presence of SB and Chel (Figure 3c).

### 3.2 Measures in isolated mitochondria

**FIGURE 4:**
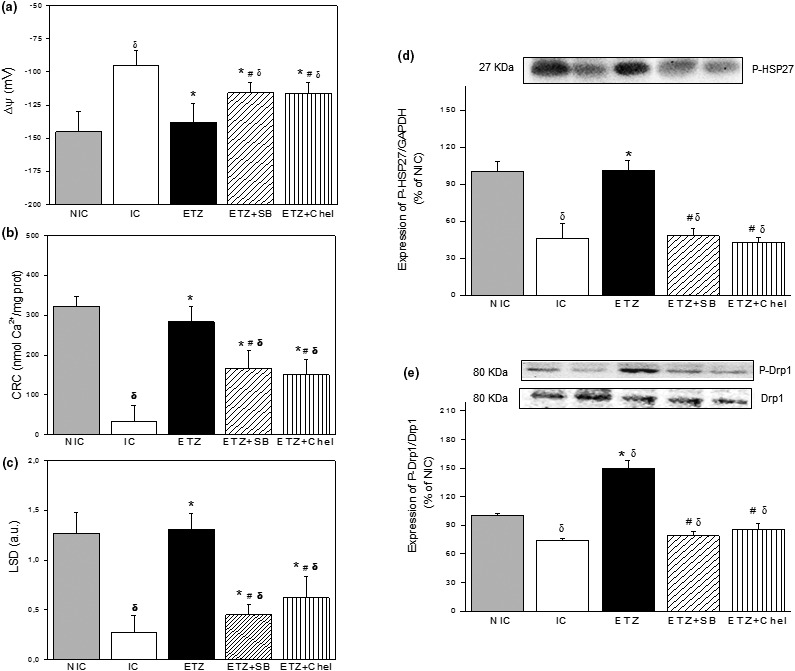
Mean values of mitochondrial potential (ΔΨm, A), calcium retention capacity (CRC, B), light scattering decrease (LSD, C), expression of P‐HSP27 referred to 3C loading control (D) and P‐Drp1/Drp1 (E) ratios of non‐ischemic (NIC, *n* = 5), ischemic control (IC, *n* = 5) hearts and in hearts treated with ETZ in absence and in presence of SB (*n* = 5) or Chel (*n* = 5). Observe that ETZ improved CRC and LSD, restored ΔΨm and increased the expression of P‐HSP27 and P‐Drp1. These changes were significantly attenuated or abolished when p38MAPK or PKC were inhibited. ^δ^ *p* < 0.05 vs. NIC; ^*^ *p* < 0.05 vs. IC; ^#^ *p* < 0.05 vs. ETZ.

Figure 4 shows the mitochondrial potential (ΔΨm, Figure 4a), the CRC (Figure 4b), and the LSD (Figure 4c) in mitochondrial samples derived from NIC, IC, ETZ, ETZ + SB, and ETZ + Chel groups. In IC hearts, ΔΨm reached lower negative values (~ −95 mV) than those detected in NIC hearts (~ −145 mV) and exhibited a marked reduction in CRC and LSD (80%–90%) compared to the NIC group. ETZ treatment normalized these parameters reaching similar values of ΔΨm, CRC, and LSD to those obtained in NIC hearts. These ETZ‐mediated beneficial effects were significantly attenuated when p38MAPK and PKCε were inhibited.

The content of the phosphorylated form of HSP27 decreased in IC hearts. This reduction was not detected in ETZ‐treated hearts and was again observed when ETZ was administered in the presence of SB or Chel (Figure 4d). Similar changes were observed in the content of PSer637 Drp1. Thus, the content of P‐Drp1 decreased in IC hearts and this reduction was absent in ETZ‐treated hearts reaching higher values than that NIC hearts. This beneficial effect of ETZ was abolished when p38MAPK and PKC were inhibited (Figure 4e).

## 4 DISCUSSION

Our results demonstrate that blockade of both extracellularly oriented and intracellular CAs with ETZ limits infarct size and attenuates the myocardial and mitochondrial alterations induced by ischemia‐reperfusion. These beneficial actions appear mediated by p38MAPK‐ and PKCε‐dependent pathways.

In heart failure rats, it was demonstrated that ETZ and the poorly diffusible cell membrane benzolamide, have comparable effects in improving cardiac function and preventing left ventricular remodeling (Vargas et al., 2016). However, in an “ex vivo” model of rat hearts subjected to myocardial infarction, the reduction in infarct size of roughly 60% by ETZ was less than the 80% observed with benzolamide (Ciocci Pardo et al., 2018; González Arbeláez et al., 2018). This result was unexpected considering that the access intracellular CA by ETZ and lack of it by benzolamide (Swenson et al., 1993) should give greater protection. Thus, we must conclude that the high permeability of ETZ does not significantly contribute to the increased tolerance of myocardium to ischemia‐reperfusion over and above inhibition of extracellularly oriented CA activity. Potential explanations for this unexpected response include (1) no true difference due to large enough confidence intervals around the mean or (2) inhibition of intracellular CA isozymes with unknown downstream effects (such as greater intracellular acidosis) may blunt the effect of selective inhibition of extracellularly oriented CAs. In any event, both inhibitors appear protective and suggest that acetazolamide, which is intermediate in diffusibility between ETZ and benzolamide, and is clinically available might be protective in patients presenting with acute myocardial infarction undergoing revascularization.

During ischemia, the increased generation of H^+^ (fall in intracellular pH) leads to the activation of Na^+^‐H^+^ exchanger isoform 1 (NHE1) and Na^+^‐${\text{HCO}}_{3}^{‐}$ co‐transport (NBC) as alkalinizing mechanisms that result in an increased intracellular Na^+^ concentration (Vaughan‐Jones et al., 2009). This rise in Na^+^, coupled with the membrane depolarization, results in a reversal of the normal extrusion of intracellular Ca^2+^ by the Na^+^/Ca^2+^ exchanger to bring Ca^2+^ into the cardiomyocyte. During reperfusion, these events are further magnified leading to more intracellular Ca^2+^ overloading simultaneously with a restoration of normal pHi (Pittas et al., 2018). As NBC and NHE1 are associated with CA, inhibition of CA with ETZ will reduce the activity of both transporters by limiting their substrate availability. Furthermore, because CA activity also facilitates CO_2_ removal, which accumulates during ischemia from buffering of intracellular lactic acid, the maintenance at the onset of reperfusion of ischemic acidic pH and the concomitant decrease in intracellular Na^+^ and Ca^2+^ concentration could be the first consequences of CA inhibition with ETZ. Both events, a diminution of Ca^2+^ concentration and the maintenance of acid pH have been also associated with the recently reported beneficial effects obtained by blockade of the electrogenic isoform of NBC (Ciocci Pardo et al., 2019). On the other hand, although the ${\text{HCO}}_{3}^{‐}$ is necessary for redox reactions and mitochondrial metabolism, it was previously demonstrated (Queliconi et al., 2016) that a high concentration of this anion increases induced reperfusion damage. Since the blockade of CA leads to less activity of ${\text{HCO}}_{3}^{‐}$‐dependent transporters, less ${\text{HCO}}_{3}^{‐}$ influx could be occurring in our experimental conditions. Therefore, this action could be contributing to the beneficial effects of ETZ.

The role of p38MAPK in myocardial ischemic injury is particularly controversial, with some studies showing that its activation accentuates the injury and other suggesting that it can be cardioprotective (Kumphune et al., 2012). In this study, an increase in p38MAPK level was detected in ETZ‐treated hearts. Moreover, the p38MAPK inhibition abolished the ETZ‐mediated cardioprotection. These results demonstrate the beneficial role played by p38MAPK in the pathways of protection afforded by ETZ. Which are the mechanisms responsible for p38MAPK activation? It has been previously demonstrated that p38MAPK can be activated by intracellular acidosis (Zheng et al., 2005) and inactivated by calcineurin (Lim et al., 2001). In our conditions, the ETZ‐treated hearts showed low levels of calcineurin. Therefore, the maintenance of acidosis and inactivation of calcineurin by diminished Ca^2+^ uptake can be implicated in the ETZ‐mediated increase in p38MAPK expression.

Protein kinases C (PKCs) constitute a family of serine/threonine kinases with a broad tissue distribution, with PKCε the principal isoenzyme expressed in rat heart (Bogoyevitch et al., 1993). This isoform of PKC plays a key role in cardioprotection (Inagaki et al., 2006). Herein, we show that the reduction in P‐PKCε detected in non‐treated hearts was avoided by ETZ treatment suggesting the involvement of PKC in the ETZ‐mediated cardioprotection.

Mitochondrial Ca^2+^ overload is an important trigger of mPTP opening resulting in mitochondrial depolarization and cardiomyocyte death (Briston et al., 2019; Ong et al., 2015). In this study, mitochondria isolated from ETZ‐treated hearts displayed a reduction in membrane depolarization, an increase in mPTP Ca^2+^‐induced response and a resistance to opening. These beneficial actions on mitochondria were attenuated when p38MAPK or PKC was blocked suggesting the important contribution of both kinases to the mitochondrial protection afforded by ETZ.

On the other hand, mitochondria are dynamic organelles that maintain their morphology and function by the balance between the opposing processes of fusion and fission (Youle & van der Bliek, 2012). Increased mitochondrial fission is involved in cardiomyocyte death and deterioration in cardiac function produced by ischemia‐reperfusion (Maneechote et al., 2017). The inhibition of mitochondrial fission decreases the infarct size and improves postischemic contractility. In this process Drp1, a dynamin‐related protein‐1, plays a crucial role. When Drp1 is phosphorylated at Ser616 translocates to mitochondrial scission sites and promotes mitochondrial fragmentation. Contrarily, the activation/phosphorylation of Drp1 at Ser637 blocks mitochondrial fission. Therefore, changes in mitochondrial morphology are accompanied by Drp1 phosphorylation at different sites. In this study, IC hearts show a decrease in PSer637‐Drp1 content. This result, in absence of a direct visualization, could be indicating the existence of smaller and more divided mitochondria in those compared to nonischemic hearts. The level of PSer637‐Drp1 increased after ETZ treatment suggesting an anti‐fission action of CA blockade and the possibility to found some mitochondria with normal morphology in ETZ‐treated hearts. The content of PSer637‐Drp1 was restored when p38MAPK and PKC were inhibited, suggesting that both kinases‐dependent pathways are participating in the prevention of postischemic alterations of mitochondrial dynamic afforded by ETZ.

The data presented to date support the participation of p38MAPK and PKCε in the beneficial effects of ETZ. It has previously been described that the heat shock protein 27 kDa (HSP27) is a possible target of both protein kinases (Marais et al., 2005; Moench et al., 2009). Our data show that ETZ treatment prevented the decrease in P‐HSP27 expression observed in IC hearts. This action was abolished when both kinases were inhibited suggesting that HSP27 is one possible end target of p38MAPK‐ and PKC‐dependent pathways. What are the actions mediated by HSP27? Several studies (Efthymiou et al., 2004; McGinley et al., 2013; You et al., 2009) show that HSP27 exerts antioxidant and anti‐inflammatory actions and is able to stimulate mitochondrial activity. It was also suggested a direct interaction of HSP27 with one or more components of mPTP thus preventing the release of cytochrome C (Samali et al., 2001). These actions of HSP27 could be involved in the ETZ‐mediated attenuation of mPTP opening and fission process.

Other actions of p38MAPK and PKC not related to HSP27 could contribute to the cardioprotection afforded by ETZ. Thus, previous studies show that MAPKs affect mitochondria‐mediated cell survival and cell death through their effects on reactive oxygen species and Ca^2+^ signaling (Javadov et al., 2014). Thus, this may be one means by which the ability of p38MAPK acts to avoid mitochondrial Ca^2+^ overload. Regarding PKC, the ability of this kinase to interact with CA/Cl^−^/${\text{HCO}}_{3}^{‐}$ exchanger metabolon may thus reduce ${\text{HCO}}_{3}^{‐}$ transport (Morgan et al., 2007). Interfering with mPTP components (Baines et al., 2003) associated with calcineurin can induce the transcription of cardioprotective genes (Budas & Mochly‐Rosen, 2007; Mesquita et al., 2014), and to activate mitochondrial ATP‐dependent K^+^ channels (Garg & Hu, 2007) are other actions attributed to PKC.

It was previously demonstrated (Baines et al., 2002), that the translocation to specific subcellular compartments is an important mechanism for PKCε and MAPK to downstream signaling protective cascades. Taking into account, that cardioprotection achieved by ETZ is mediated by activation of both kinases, CAs localized in cytosol and mitochondria could be involved.

On the other hand, and considering the recent association between benefits of therapeutic hypercapnia and improved mitochondrial function (Chi et al., 2019), our conclusion about the important contribution of mitochondrial actions to cardioprotection ETZ mediated become more relevant.

## 5 CONCLUSIONS

**FIGURE 5:**
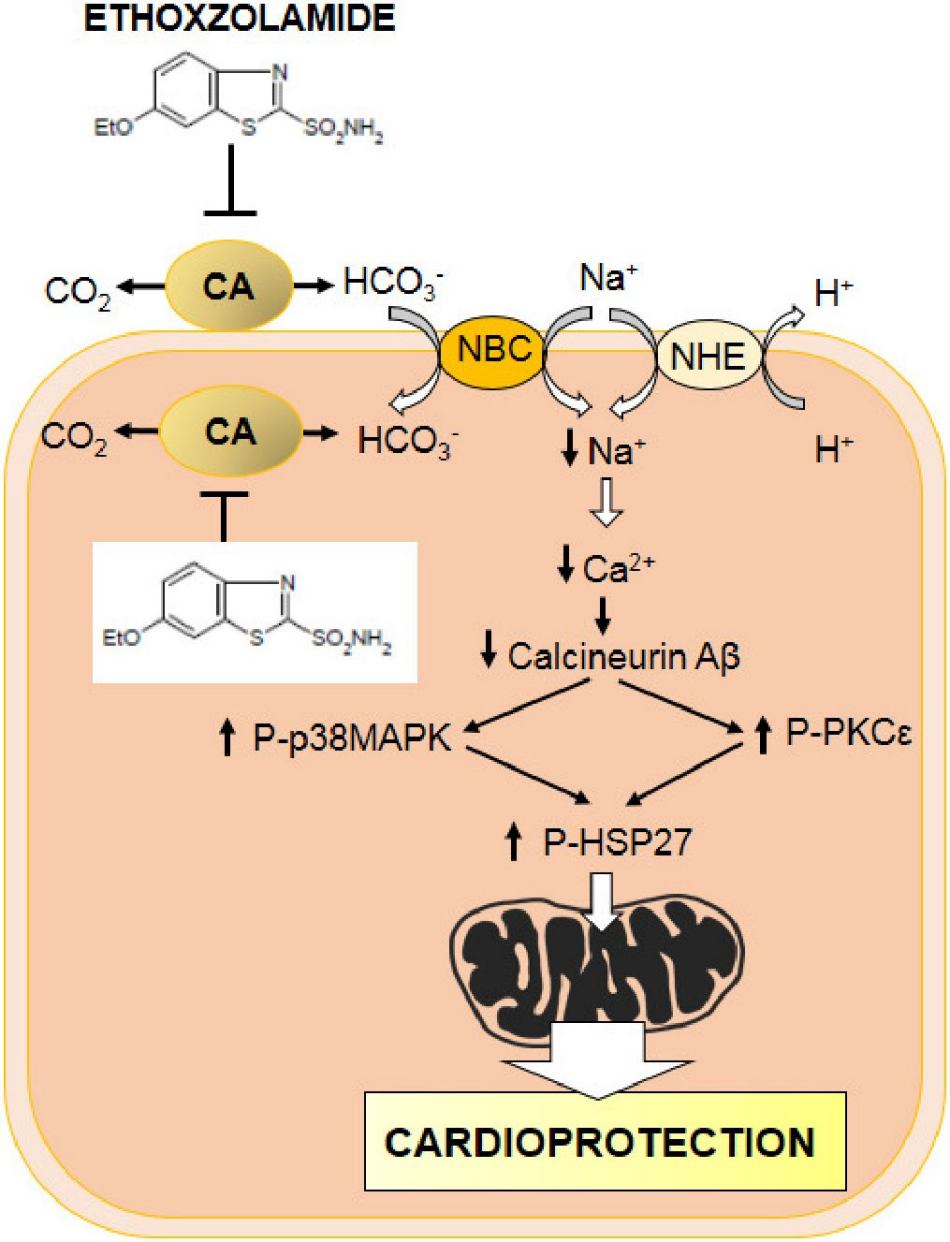
Mechanisms involved in ethoxzolamide‐mediated cardioprotection

The inhibition of CA with ETZ appears to protect the heart from ischemia‐reperfusion injury by an attenuation of mPTP formation and/or opening and mitochondrial fission. A decrease in intracellular Ca^2+^ loading consequent to the maintenance of an acid pH in the presence of ETZ could be involved in less calcineurin activation, and increased p38MAPK, PKCε, and HSP‐27 phosphorylation/activation (Figure 5). These findings reinforce the role of CA as possible target for the design of new pharmacological agents useful in the management of cardiac diseases.

## CONFLICT OF INTEREST

None.

## AUTHOR CONTRIBUTIONS

**S**.M.M. and B.V.A. conceived and designed research; A.C.P., L.F.G.A., and J.C.F. performed experiments and analyzed data; S.M.M., B.V.A., and E.R.S. interpreted results of experiments; A.C.P., L.F.G.A., and J.C.F. prepared figures; S.M.M. wrote the draft version of manuscript; E.R.S. and B.V.A. edited and revised the manuscript; S.M.M. approved the final version of manuscript.
